# Effects of Different Levels of Antarctic Krill Oil on the Ovarian Development of *Macrobrachium rosenbergii*

**DOI:** 10.3390/ani14223313

**Published:** 2024-11-18

**Authors:** Xiaochuan Zheng, Jie Yang, Xin Liu, Cunxin Sun, Qunlan Zhou, Aimin Wang, Jianming Chen, Bo Liu

**Affiliations:** 1 Key Laboratory for Genetic Breeding of Aquatic Animals and Aquaculture Biology, Freshwater Fisheries Research Center (FFRC), Chinese Academy of Fishery Sciences (CAFS), Wuxi 214081, China; zhengxiaochuan@ffrc.cn (X.Z.); 35118213@njau.edu.cn (J.Y.); 2023213007@stu.njau.edu.cn (X.L.); suncx@ffrc.cn (C.S.); zhouql@ffrc.cn (Q.Z.); 2 Wuxi Fisheries College, Nanjing Agricultural University, Wuxi 214081, China; 3 College of Marine and Biology Engineering, Yancheng Institute of Technology, Yancheng 224051, China; blueseawam@ycit.cn; 4 Key Laboratory of Fish Health and Nutrition of Zhejiang Province, Zhejiang Institute of Freshwater Fisheries, Huzhou 313001, China

**Keywords:** functional feed, ovarian histology, giant freshwater prawn, reproductive hormones, vitellogenesis

## Abstract

Antarctic krill oil has been proven to be able to promote the ovarian development of crustaceans, but its optimal application dose and potential regulatory mechanism in *Macrobrachium rosenbergii* are still unclear. In this study, five isonitrogenous and isolipidic diets with gradient additions of Antarctic krill oil (0%, 1.5%, 3%, 4.5%, and 6%) were served exposed to 8 weeks of feeding. The results show that 3–4.5% Antarctic krill oil supplementation significantly increases the weight gain rate and specific growth rate of *M. rosenbergii* (*p* < 0.05). In addition, 3–4.5% Antarctic krill oil supplementation significantly increased the content of hemolymph vitellogenin (VTG) and the levels of reproductive hormones, including methyl farnesoate (MF), estradiol (E_2_), and progesterone (P_4_) (*p* < 0.05). The differences in ovarian index, oocyte volume, yolk granule deposition in oocytes, and the transcription levels of *VTG* genes in hepatopancreas and ovarian tissues demonstrated that the addition of Antarctic krill oil significantly promoted ovarian development and vitellogenesis, especially at the 4.5% addition level. In terms of molecular signaling, this study confirms that the retinol metabolic signaling pathway, MF signaling pathway, steroid hormone signaling pathway, and ecdysone signaling pathway, along with their specific molecules, such as Farnesoic acid-O-methyltransferase (*FAMeT*), retinoid x receptor (*RXR*), ecdysone receptor (*EcR*), and estrogen-related receptor (*ERR*), are involved in the regulation of the ovarian development of *M. rosenbergii* by adding Antarctic krill oil at appropriate doses. The findings indicate that the supplementation of 4.5% Antarctic krill oil in the diet is optimal for stimulating the secretion of reproductive hormones in female *M. rosenbergii*, thereby promoting vitellogenesis and ovarian development.

## Simple Summary

The lack of in-depth research and development on specialized functional feed for broodstock gonad maturation represents a significant obstacle to the industrialization of *Macrobrachium rosenbergii* breeding. The potential of Antarctic krill oil to promote ovarian development in crustaceans has been established; however, the optimal application dose and potential regulatory mechanism in *M. rosenbergii* remain unclear. This study established a gradient addition of Antarctic krill oil (0%, 1.5%, 3%, 4.5%, and 6%) for a period of eight weeks. Antarctic krill oil supplementation in the range of 3–4.5% resulted in a significant promotion of ovarian development, vitellogenesis, and reproductive hormone synthesis. The retinol metabolic signaling pathway, methyl farnesoate (MF) signaling pathway, steroid hormone signaling pathway, and ecdysone signaling pathway along with their specific molecules were found to be involved in the regulation of ovarian development of *M. rosenbergii* with the use of Antarctic krill oil. The results demonstrate that the 4.5% supplementation Antarctic krill oil in the diet is optimal for stimulating the secretion of reproductive hormones in female *M. rosenbergii*, thereby promoting vitellogenesis and ovarian development.

## 1. Introduction

*Macrobrachium rosenbergii* is the world’s largest prawn, native to South Asia and Southeast Asia, as well as northern Oceania and the Western Pacific islands [1]. In 2022, the global production of *M. rosenbergii* reached about 230,000 metric tons, which has been growing continuously in recent years [2]. In 2023, the production of *M. rosenbergii* in China will reach 196,000 tons, accounting for more than 50% of global aquaculture production. In recent years, the rapid expansion of the aquaculture area has posed a challenge to the stable supply of *M. rosenbergii* larvae. However, at present, some prominent problems need to be addressed in the process of artificial seedling breeding, such as insufficient broodstock, poor gonad maturation, and the relatively unconcentrated oviposition caused by the reproductive dysfunction of broodstock, which leads to unstable breeding, a prolonged breeding cycle, and increased production costs [3]. The immature, artificial, large-scale, seedling breeding system and the inadequate research and development of special functional feed for broodstock gonad maturation have been obstacles restricting the industrialization progress of *M. rosenbergii* breeding. Therefore, exploring effective methods for the induction of gonad maturation is urgently needed for *M. rosenbergii* culture and larval production. The precise regulation of broodstock reproduction using a nutrition strategy is an economical and effective method, which mainly involves the use of protein, lipids, or vitamin-based nutrients. These nutrients not only provide energy for broodstock prawns, but also act as precursors of important reproductive hormones to regulate the secretion of hormones, gonadal development ability, and fecundity [4,5,6].

Antarctic krill oil is a lipid extract extracted from Antarctic krill whole shrimp or krill powder. The current technologies employed for the extraction of krill oil encompass a range of techniques, including solvent extraction, non-solvent extraction, supercritical/subcritical fluid extraction, and enzyme-assisted pretreatment extraction [7,8,9]. Krill oil is a valuable source of nutrients for aquatic animals, providing a rich array of lipids, including phospholipids, triacylglycerol, and other essential compounds. Additionally, krill oil contains trace elements, such as astaxanthin, tocopherol, vitamin A, and cholesterol, which contribute to its nutritional profile [10,11]. It is estimated that 30–65% of omega-3 polyunsaturated fatty acids (PUFAs) present in krill oil are stored in the form of phospholipids [12]. The principal constituents of prawns’ ovarian tissue are highly unsaturated fatty acids (HUFAs) and phospholipids (PLs). However, crustaceans have traditionally been regarded as having a limited ability to synthesize these compounds to a sufficient extent to meet the nutritional requirements for gonadal maturation [13]. It is therefore necessary to supplement the feed of prawn broodstock with phospholipid sources to meet their nutritional requirements for gonadal maturation. The appropriate amount of PLs is essential for the normal development of oocytes in crustaceans, while excessive or insufficient PL nutrition may reduce their reproductive capacity. Insufficient lipid nutrition makes it challenging to maintain normal reproductive function [14]. Excessive PL nutrition can increase the possibility of phospholipid peroxidation and the level of reactive oxygen species, thus hindering the growth and development of aquatic animals [15,16,17]. Consequently, a further investigation of the impact of feeding different levels of Antarctic krill oil on the ovarian development of *M. rosenbergii* and the regulatory mechanisms governing ovarian development is warranted.

The oogenesis process of *M. rosenbergii* can be broadly delineated into five stages: the oogonia, the pre-vitellogenic oocytes, the endogenous vitellogenic oocytes, the exogenous vitellogenic oocytes, and the mature oocytes [18]. Vitellogenesis represents a pivotal process and a crucial marker of ovarian development and maturation for female crustaceans. It refers to the synthesis of yolk substances (including proteins, lipids, and carbohydrates) and their deposition in oocytes, which ensures that oocytes can provide sufficient nutrition for embryonic development. The vitellogenesis of crustaceans is mainly regulated by a variety of hormones and molecular mechanisms, including a juvenile hormone (methyl farnesoate (MF)) and steroid hormones (ecdysone and sex steroids) [19,20,21,22,23]. Laufer et al. suggest that MF secretion is associated with female reproduction, as they found that the highest MF secretion rates in *Libinia emarginata* ovaries occurred at the peak of oocyte growth and vitellogenesis [19]. A study has demonstrated that the ecdysone receptor (*EcR*) is capable of stimulating vitellogenesis through binding to HRE in the 5′ regulatory sequence of the gonad-inhibiting hormone (GIH) [24]. Summavielle et al. showed that endogenous 17β-estradiol (17β-E_2_), progesterone (P_4_), and other steroid hormones can promote the formation of yolk in *Penaeus japonicus* [25]. It can be reasonably proposed that the regulation of ovarian development and maturation of *M. rosenbergii*, to influence the synthesis of endogenous hormones and related molecules, may prove an efficacious strategy for the development of gonadal maturation regulators in *M. rosenbergii*.

In recent years, the efficacy of krill oil in regulating ovarian development has been confirmed. Xu et al. investigated the effects of three different phospholipid sources (soybean lecithin, egg yolk lecithin, and krill oil) on the ovarian development of *Cherax quadricarinatus* [26]. The study demonstrated that Antarctic krill oil supplementation significantly increased the gonadotropic index and promoted triglycerides and cholesterol deposition in the ovary and hepatopancreas. Additionally, Liang et al. investigated the effects of these three phospholipid sources. It was found that krill oil could promote the ovarian development of *Litopenaeus vannamei* by affecting the metabolism of certain key fatty acids and increasing the secretion of estradiol (E_2_) and MF [27]. On this basis, Liang et al. conducted further investigations into the optimal addition of krill oil to the diet of *L. vannamei* [28]. The results of these studies confirmed that the addition of 6% krill oil to the diet resulted in a positive effect on the ovarian development of female *L. vannamei*, as evidenced by a significant increase in the gonadosomatic index and the level of MF in females, as well as the promotion of lipid accumulation in the hepatopancreas and oocytes.

In light of the aforementioned findings, a series of experimental diets was formulated with varying concentrations of Antarctic krill oil (0%, 1.5%, 3%, 4.5%, and 6%) with the objective of examining the impact of Antarctic krill oil on the development of oocytes in *M. rosenbergii*, the synthesis of vitellogenin in the hepatopancreas and oocytes, as well as the synthesis of related reproductive hormones. This will enable us to ascertain the efficacy of Antarctic krill oil, identify the optimal concentration, and elucidate the underlying regulatory pathways. The findings of this study provide a theoretical foundation for an effective dietary intervention to promote ovarian maturation in *M. rosenbergii*, thus having the potential to contribute to the industrialization of *M. rosenbergii* artificial-scale nurseries.

## 2. Materials and Methods

### 2.1. Ethics Statement

All experiments were conducted in accordance with the guidelines for scientific breeding and use of prawns set forth by the Animal Care and Use Committee of the Ethics of Animal Experiments of Freshwater Fisheries Research Center.

### 2.2. Diet and Prawn Management

In this study, protein sources comprised a fish meal, soybean meal, cottonseed protein, spray-dried blood cell powder, and corn gluten meal. The lipid sources were soybean oil and Antarctic krill oil, while the carbohydrate sources were mainly provided by α-starch. Five groups of isonitrogenous and isolipidic experimental diets were prepared according to the nutritional requirements of *M. rosenbergii*. Different levels of Antarctic krill oil (K2:1.5%, K3:3%, K4:4.5%, and K5:6%) were added to the feed, with an Antarctic krill oil-free diet serving as the control group (K1). The lipid content of the feed was balanced by the incorporation of soybean oil. The Antarctic krill oil was procured from the Borun Laboratory Equipment Management Department (Qingdao, Shandong, China). The experimental diets were prepared in accordance with the laboratory preparation scheme and naturally dried before being stored at −20 °C. The ingredients and proximal composition of the experimental diets are presented in Table 1.

The experimental prawns were sourced from the *M. rosenbergii* original seed farm of the Zhejiang Freshwater Fisheries Research Institute, Huzhou, China, and the breeding experiment was conducted at the Dapu Base of Freshwater Fisheries Research Center of Chinese Academy of Fishery Sciences. After one week of acclimatization, 20 prawns were randomly chosen for the measurement of the initial ovarian index (OSI, 0.08 ± 0.01%).

And then 400 robust *M. rosenbergii* with an initial uniform body weight (4.56 ± 0.13 g) and the same ovary maturation status were randomly divided into 5 groups with 4 replicates per treatment and 20 prawns per replicate (inner diameter × water depth: 2.0 m × 0.8 m). To provide shelter, plastic tubes and plastic water plants were placed at the bottom of the culture bucket. During the experiment, the prawns were fed twice daily at 8:00 and 18:00 for a period of 8 weeks, with the quantity fed being in the range of 3–5% of the body weight. The residual bait amount was observed after 30 min of feeding, and the feeding amount was adjusted accordingly. The water temperature was maintained within the range of 26–31 °C, with nitrite levels ≤ 0.02 mg·L^−1^, ammonia levels ≤ 0.2 mg·L^−1^, dissolved oxygen ≥ 8 mg·L^−1^, and pH fluctuating between 7.3 and 7.8.

### 2.3. Sample Collection

After an eight-week feeding period, euthanasia was performed with MS-222 (Millipore Sigma, St. Louis, MO, USA). The weight gain rate (WGR), specific growth rate (SGR), hepatopancreatic index (HSI), and ovarian index (OSI) were calculated using the following formulas: WGR (%) = (Final weight − Initial weight)/initial weight × 100; SGR (%) = (Ln final weight − Ln initial weight)/days × 100; HSI (%) = (Hepatopancreas weight/Final weight) × 100; OSI (%) = (Ovary weight/Final weight) × 100.

Following the feeding trial, 0.5 mL of hemolymph was extracted from the pericardial cavity using a sterile 1 mL syringe, mixed at a 1:1 ratio with a pre-inhaled pre-cooled anticoagulant solution, and immediately subjected to centrifugation at 4000 rpm and 4 °C for 10 min. The resulting supernatant was then collected and stored at −20 °C for the subsequent determination of biochemical indicators. Meanwhile, the sampled prawns were dissected, and the samples of hepatopancreas and ovary were collected and immediately frozen in liquid nitrogen and stored at −80 °C for gene expression analysis.

### 2.4. Histological Analysis of Ovaries

The ovarian tissue sections of *M. rosenbergii* were fixed in 4% paraformaldehyde for a period of 24 h, subsequently dehydrated using a gradient ethanol and xylene solution, embedded in paraffin, and cut into 4 μm thick sections. The sections were then dewaxed, hydrated, and stained with hematoxylin and eosin (H&E). The tissue sections were observed under a light microscope (Olympus BX51, Tokyo, Japan). Three individuals were analyzed from each group. Only dominant oocytes with nuclei were used for the measurement of oocyte sizes. The long diameter of an oocyte (LO) and the shorter diameter of an oocyte (SO) were measured using digital pathology reading software (Shenzhen Shengqiang Technology Co., Ltd., Shenzhen, China). The volume of the oocyte (VO) was then calculated using the following formula: VO = 0.523 × W^2^ × L (W, maximum width; L, maximum length).

### 2.5. Measurements of Reproductive Hormones and Vitellogenin Concentrations in Hemolymph

Eight samples were obtained from each group for the purpose of detecting vitellogenin (VTG), ecdysone (EH), estradiol (E_2_), and progesterone (P_4_). The kit was procured from Beijing Winter Song Boye Biotechnology Co., Ltd. (Beijing, China). The detection method was carried out in accordance with the kit instructions (Shrimp VTG ELISA Kit, lot: DG91159Q; Ecdysone ELISA Kit, lot: DG91160Q; E_2_ ELISA Kit, lot: DG96556Q; P_4_ ELISA Kit, lot: DG96554Q).

### 2.6. Determination of Methyl MF Content in Hemolymph

A 1 mL sample of hemolymph from *M. rosenbergii* was combined with a 1 mL solution of 0.9% NaCl and 1 mL of acetonitrile. The mixture was vortexed for an additional minute. The solution was then centrifuged at 3000 r·min^−1^ for 5 min, and the upper organic phase was collected. The extraction was repeated twice, the combined solution was evaporated to dryness by rotary evaporation, and then the resulting n-hexane was diluted to 100 μL for GC/MS (Agilent Technologies, 7890B, PEGASUS BT, Wilmington, DE, USA) analysis.

### 2.7. Quantitative Real-Time PCR

Total RNA was extracted from intestinal tissues of five experimental groups (eight samples in each group) using the RNAiso Plus reagent (Takara, Dalian, China). The RNA concentration was determined by measuring the absorbance at a wavelength of 260:280 nm (OD260/OD280 = 1.8–2.0) by using a Nanodrop 2000 (NanoDrop Technologies Co., Ltd., Wilmington, DE, USA). First-strand cDNA was synthesized for qRT-PCR analysis using the HiScript^®^ III RT SuperMix (+gDNA wiper) (Vazyme, Nanjing, China). The qRT-PCR was conducted with TB Green^®^ Premix Ex Taq™ II (Tli RNaseH Plus) (Takara, Dalian, China), in accordance with the manufacturer’s instructions. The *18S rRNA* gene was selected as the reference gene, and each sample was tested in triplicate. Online design tools (NCBI, Bethesda, MA, USA) were employed for primer design (Table 2). The CDS sequence for designing the selected gene primers was obtained from our ovary transcriptome sequencing database of *M. rosenbergii* (National Institutes of Health’s Short Read Archive database accession no. PRJNA1186241). The results were calculated using the 2^−ΔΔCt^ method and the *18S rRNA* was selected as the housekeeping gene to normalize our samples because of its stable expression in the present study. The gene-specific primers were synthesized by Shanghai Generay Biotech Co., Ltd. (Shanghai, China).

### 2.8. Statistical Analysis

The experimental data were subjected to analysis using SPSS 20.0 software (SPSS Inc., Chicago, IL, USA). Levene’s test was employed to assess the normality of the data and the homogeneity of variance. Subsequently, a one-way analysis of variance (One-Way ANOVA) and Tukey’s honest significant difference (Tukey’s HSD) test were conducted to ascertain the existence of statistically significant differences in the indicators between the five groups. All data were expressed as mean ± standard error (X ± SEM). *p* < 0.05 was considered statistically significant. The orthogonal polynomial method was used to perform linear and quadratic regression analyses on the impact of varying levels of Antarctic krill oil supplementation on the outcome variable. The level of significance was set at *p* < 0.05 to determine the extent of any observed differences.

## 3. Results

### 3.1. Effects of Antarctic Krill Oil on the Growth Performance of M. rosenbergii

The growth performance of *M. rosenbergii* after adding different doses of krill oil to the feed is shown in Table 3. Compared with the K1 group, the mean final weight (FW), weight gain rate (WG), and specific growth rate (SGR) of *M. rosenbergii* in the K3 and K4 groups were significantly increased (*p* < 0.05). The highest values were found in the K4 group (*p* < 0.05). FW, WGR, and SGR levels showed primary linear (*p* < 0.05) and quadratic (*p* < 0.05) changes with increasing levels of krill oil. No statistically significant differences in survival rates (SRs) were observed among the groups (*p* > 0.05).

### 3.2. Effects of Antarctic Krill Oil on the Hepatopancreas Index and Ovary Index of M. rosenbergii

**Figure 1:**
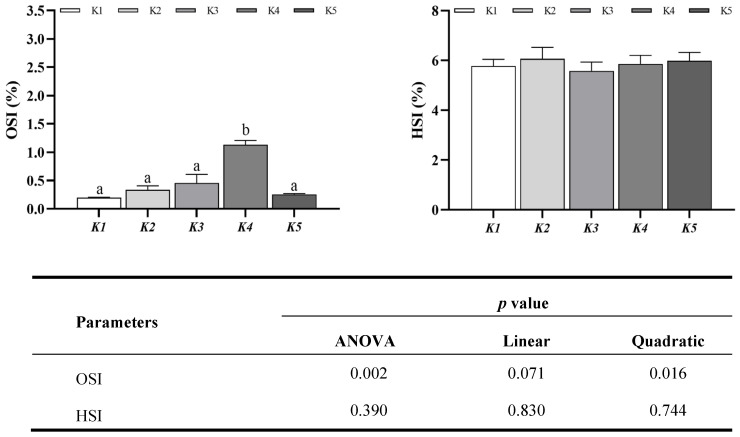
Effect of different levels of krill oil on the hepatosomatic index (HSI) and ovary index (OSI) in *Macrobrachium rosenbergii.* Each datum represents the mean of twenty replicates. Bars assigned different superscripts are significantly different.

As shown in Figure 1, as far as the ovarian index is concerned, compared with K1, the ovarian index of *M. rosenbergii* in the K4 group is significantly increased (*p* < 0.05), and there is no significant difference in the ovarian index of *M. rosenbergii* in the K2, K3, and K5 groups (*p* > 0.05). The changes in the ovarian index of *M. rosenbergii* were quadratic (Quadratic, *p* < 0.05) with increasing levels of Antarctic krill oil. For the hepatopancreatic index, there was no statistically significant difference (*p* > 0.05) in the hepatopancreatic index between the groups of *M. rosenbergii*.

### 3.3. Effects of Antarctic Krill Oil on the Ovarian Histomorphology and Oocyte Parameters of M. rosenbergii

**Figure 2:**
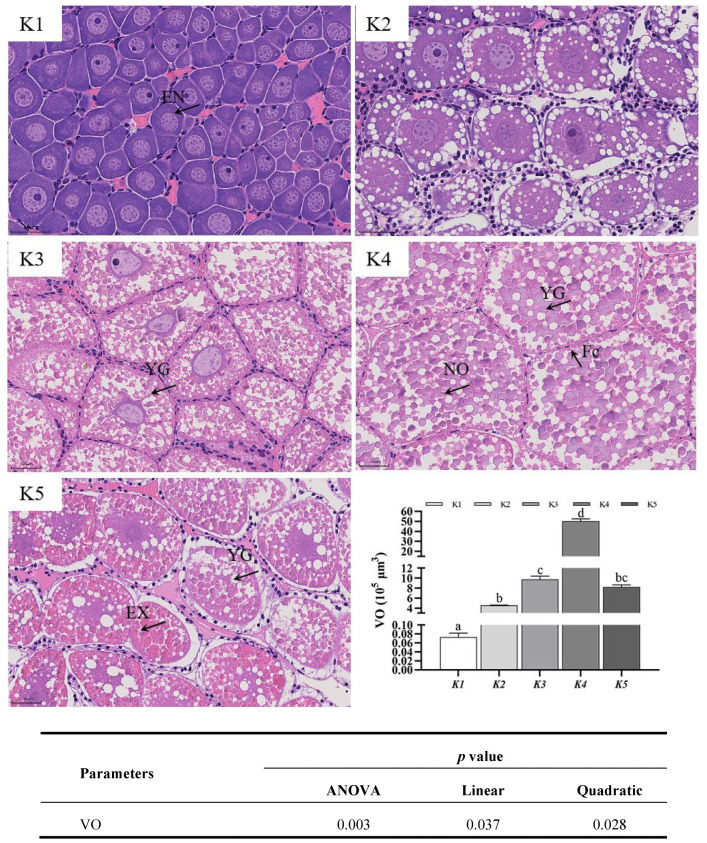
H&E staining of ovarian tissue from *Macrobrachium rosenbergii* subjected to dietary Antarctic krill oil. Eight replicates were performed for each group. EN, endogenous vitellogenic oocyte; EX, exogenous vitellogenic oocyte; NO, nearly mature oocyte; FC, follicle cell; YG, yolk granule; Photomicrographs (40) with scale bars (50 μm). Bars assigned different superscripts (a–d) are significantly different.

As shown in Figure 2, a large number of basophilic endogenous vitellogenesis-stage oocytes is present in the ovarian tissues of groups K1 and K2, and a large number of eosinophilic exogenous vitellogenesis-stage oocytes with many eosinophilic yolk granules (YGs) is present in the cytoplasm of the ovarian tissues of groups K3, K4, and K5. At the same magnification of the light microscopic field of view (40×), group K4 had the lowest number of oocytes and the largest YGs of oocyte cytoplasmic deposition. Oocyte volume was significantly higher in groups K2, K3, K4, and K5 compared to group K1 (*p* < 0.05). The highest values were found in group K4 (*p* < 0.05). Oocyte volume increased linearly (*p* < 0.05) and quadratically (*p* < 0.05) with the addition of Antarctic krill oil.

### 3.4. Effects of Antarctic Krill Oil on the Reproductive Hormone Secretion Level of M. rosenbergii

As shown in Table 4, compared with control group K1, the VTG and E_2_ levels are significantly higher in groups K3, K4, and K5 (*p* < 0.05); EH levels are significantly higher in groups K2, K3, K4, and K5 (*p* < 0.05); and P_4_ and MF levels are significantly higher in groups K3 and K4 (*p* < 0.05). VTG, E_2_, and P_4_ levels varied linearly (*p* < 0.05), EH levels varied linearly (*p* < 0.05) and quadratically (*p* < 0.05), and MF levels varied quadratically (*p* < 0.05). The highest values of all five indices were found in the K4 group (*p* < 0.05).

### 3.5. Effects of Antarctic Krill Oil on the Expression of Vitellogenin, Vitellogenin Receptor Gene, and Maturation Promoting Factor in M. rosenbergii

**Figure 3:**
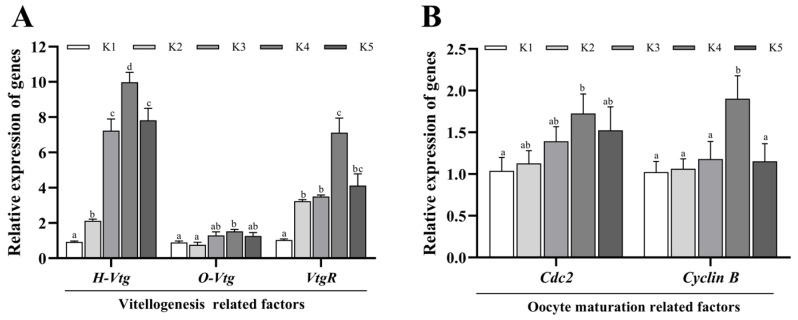
Effects of krill oil on vitellogenesis and oocyte maturation-related factors in *Macrobrachium rosenbergii*. Each datum represents the mean of twelve replicates. Bars assigned different superscripts are significantly different. (**A**). The relative expression of genes associated with vitellogenesis. (**B**). The relative expression of genes associated with oocyte maturation.

As illustrated in Figure 3, it is evident that, when the quantity of krill oil supplementation is within the range of 4.5% or below, the transcription level of *Vtg* in the hepatopancreas exhibits a notable increase with the augmentation of krill oil supplementation (*p* < 0.05). This pattern was observed up until the point where the addition amount reached 6%. A significant increase in the transcription level of *Vtg*, *Cdc2*, and *Cyclin B* in the ovary of the K4 group was observed in comparison to control group K1 (*p* < 0.05). Compared with control group K1, the transcription levels of *VtgR* in the ovaries of the K2, K3, K4, and K5 groups were significantly increased (*p* < 0.05), with the highest level observed in the K4 group.

### 3.6. Effects of Antarctic Krill Oil on Ovarian Development-Related Genes in M. rosenbergii

**Figure 4:**
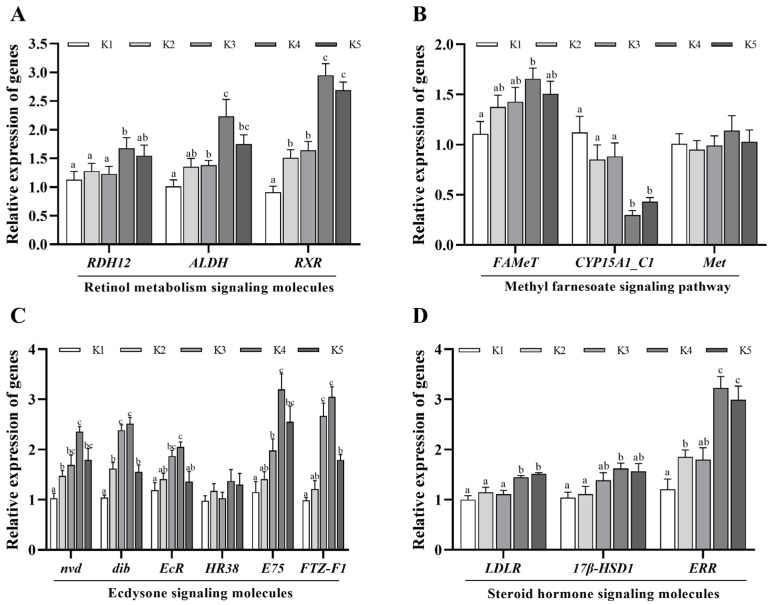
Effects of Antarctic krill oil on ovarian development-related signaling molecules in *Macrobrachium rosenbergii*. Each datum represents the mean of twelve replicates. Bars assigned different superscripts are significantly different. (**A**). The relative expression of genes associated with the retinol metabolism signal pathway. (**B**). The relative expression of genes associated with the methyl farnesoate signal pathway. (**C**). The relative expression of genes associated with the ecdysone signal pathway. (**D**). The relative expression of genes associated with the steroid hormone signal pathway.

As illustrated in Figure 4, for retinol metabolic signaling molecules, a significant increase in *RDH12* mRNA can be observed in the K4 group compared to the K1 group (*p* < 0.05). No significant effect was observed in the other groups. Concurrently, the expression levels of *ALDH* and *RXR* mRNA in the diet supplemented with krill oil were found to be significantly elevated (*p* < 0.05) in comparison to the K1 group, with the K4 group exhibiting the highest expression level. When compared to the K1 group, the expression of *FAMeT* mRNA in K4 group was found to be significantly increased (*p* < 0.05), while the expression of *CYP15A1_C1* mRNA was significantly decreased (*p* < 0.05). The addition of krill oil had no discernible effect on the expression of *Met*. In the case of ecdysone signaling molecules, the expressions of *nvd* and *dib* mRNA in the K2, K3, K4, and K5 groups, which were supplemented with krill oil, were found to be significantly increased in comparison to the K1 group, (*p* < 0.05). The expression levels of *EcR*, *E75*, and *FTZ-F1* mRNA in K3, K4, and K5 groups were significantly increased (*p* < 0.05), whereas no significant difference was observed in the K2 group. The overall trend exhibited an initial increase, followed by a subsequent decrease. The expression levels of each gene in the K4 group were the highest. The addition of krill oil did not affect the expression of *HR38*. With regard to steroid hormone signaling molecules, the expression levels of *LDLR*, *17β-HSD1*, and *ERR* mRNA in the K4 and K5 groups, which comprised a high proportion of krill oil, were significantly increased in comparison to control group K1 (*p* < 0.05). Conversely, the addition of a low proportion of krill oil had no significant effect on these genes.

## 4. Discussion

The Antarctic krill is a rich biological resource, and the krill oil extracted from it is of high nutritional value [35,36] with significant market development potential. As China’s Antarctic krill oil extraction and concentration processing systems continue to be upgraded, and as the nutritional value and application fields of Antarctic krill oil continuously expand, it is anticipated that the cost of Antarctic krill oil will decrease gradually. It will become an economically viable aquatic feed additive, particularly during specific growth stages of aquatic animals. As an efficient nutritional feed additive, krill oil plays an important role in the growth and development of crustaceans. Studies have shown that krill oil as a lipid source of *Macrobrachium nipponense* feed shows better potential than fish oil and soybean oil in improving its growth performance [37]. In this experiment, the addition of 3% and 4.5% of Antarctic krill oil significantly improved the growth performance of *M. rosenbergii*, while the addition of 6% showed a downward trend compared with the K4 group. This growth difference may be caused by the increase in ω-3 HUFA content and ω-3/ω-6 ratio with the increase in the Antarctic krill oil addition ratio [14], and the excessive ω-3/ω-6 ratio hindered the growth of *M. rosenbergii*. The study of Lv et al. also pointed out that the growth performance of *M. rosenbergii* increased first and then decreased with the increase in the dietary ω-3PUFA/ω-6PUFA ratio [38]. This suggests that an optimal dosage of krill oil can enhance the growth and development of *M. rosenbergii*; however unsuitable krill oil supplementation may impose constraints on its growth potential.

Like other crustaceans, the ovarian development of *M. rosenbergii* is roughly divided into three stages, namely, the slow growth stage, the rapid development stage, and the stable stage. During the rapid development stage, the HSI of *M. rosenbergii* decreased, while the OSI increased sharply. The study of Chen et al. showed that the mean value of the OSI at the ovarian mature stage of *M. rosenbergii* was in the range of 0.77–5.70% [39]. The study of Crisp et al. on *Metapenaeus dalli* also showed that the average OSI of the object was 0.58% at the immature stage, the maximum value was 6.90% at maturity, and it decreased sharply to 1.97% after spawning [40]. Therefore, the HSI and OSI are the key indicators to distinguish the development stage of crustacean and judge the maturity of the ovary [41,42]. In this experiment, it was observed that the OSI of the K4 group was markedly elevated in comparison with other groups, and there was a gentle decline in the HSI, indicating that the K4 group was in the stage of rapid ovarian development, while other groups were observed to display relatively delayed developmental processes. The reason may be that, in the rapid development stage, the exogenous VTG synthesized in the hepatopancreas is transported to the ovary through the hemolymph, and directly or indirectly enters the developing oocytes to form vitellin and accumulate [43,44], prompting a sharp increase in oocyte volume [45]. There was a large number of yolk granules in the ovarian tissue of the K4 group. The oocyte space in the K4 group was smaller, irregularly shaped, and polygonal, indicating that the addition of 4.5% Antarctic krill oil promoted the development of *M. rosenbergii oocytes*. The cell volume increased and the cells squeezed each other. This is consistent with the significantly increased mRNA expression levels of *H-TVG*, *O-VTG*, and *VtgR* in this study. The significant increase in *VtgR* confirmed that the transport of *VTG* is in a vigorous period. It was also observed that the mRNA expression levels of *Cdc2* and *CyclinB* genes were significantly upregulated in the K4 group compared to other groups. *Cdc2* is a major regulator of the cell cycle, and its activity depends on the presence of *CyclinB*. The combination of the two results in the formation of the M-phase promoting factor (MPF, an oocyte maturation promoting factor) [46]. The increase in these expression levels of *Cdc2* and *cyclinB* is considered to be an indicator of oocyte developmental competence [47]. In naturally mature female prawns, the expression levels of *Cdc2* and *cyclinB* increase with ovarian maturity. For example, it has been shown that the expression level of *Cdc2* increases significantly at different stages of ovarian development (e.g., stage I to stage IV), especially in stage IV (mature) ovaries [48]. This again confirmed that 4.5% Antarctic krill oil can promote the ovarian development and vitellogenesis of *M. rosenbergii*. However, it is worth noting that the high dose of Antarctic krill oil (K5 group) did not play a role in promoting these factors. Similar results were also observed for *Portunus trituberculatus* [49] and *Pangasianodon hypophthalmus* [17]. It is speculated that, on the one hand, the supplementation of more-than-appropriate levels of PL may increase the secretion of gonadal inhibitory hormones and yolk-formation inhibitory hormones [27]. On the other hand, different proportions of Antarctic krill oil will lead to changes in fatty acid composition. The development and maturation of oocytes depend on the acquisition of essential components, including fatty acids. If the parent organism has appropriate fatty acids, they will be transferred from the body tissue to the ovary [50]. Therefore, the appropriate proportion of fatty acids promotes ovarian development, while the inappropriate proportion of fatty acids has a negative impact on ovarian development.

Phospholipids have been shown to promote ovarian development in crustaceans by regulating the expression of *VTG* at the transcriptional level and promoting the transfer of VTG from the hepatopancreas to the ovary [51]. The process of vitellogenesis is regulated by different reproductive hormones and related signaling pathways, including those involved in the signaling of sex steroids, ecdysone, the MF, and retinol metabolism [52,53]. In light of the aforementioned information, we explored the response of four hormone signaling pathways to Antarctic krill oil intake from biochemical and molecular transcription levels (synthetic/metabolic enzymes and related receptors). The objective was to systematically and comprehensively evaluate the effects of Antarctic krill oil on the reproductive endocrine of *M. rosenbergii* and its associated molecular pathways.

With regard to sex steroid hormone signaling pathways, an evaluation was conducted of the contents of E_2_ and P_4_, as well as the gene expression of *LDLR*, *17β-HSD*, and *ERR*. Cholesterol serves as a substrate for steroid hormone synthesis, which is typically absorbed and transported to oocytes via *LDLR*-mediated endocytosis [54]. 17β-hydroxysteroid dehydrogenase (*17β-HSD*) represents a rate-limiting enzyme in the synthesis of steroid hormones. It participates in the final step of cholesterol synthesis steroids and plays a role in controlling animal E_2_ concentration [55]. *ERR* is an E_2_-related receptor that regulates ovarian development in *P. trituberculatus* by regulating oocyte meiosis and the synthesis and release of reproductive-related hormones [56]. The results of this study indicate that the contents of E_2_ and P_4_ in the ovaries of *M. rosenbergii* in the K3 and K4 groups were significantly increased, as was the expression of sex steroid hormone synthesis-related genes in K4. These findings suggest that 4.5% Antarctic krill oil can stimulate the synthesis of steroid hormones in *M. rosenbergii*. Sex steroid hormones can induce crustaceans to synthesize VTG and promote the proliferation and differentiation of follicular cells, which is conducive to ovarian development. It has been demonstrated that phospholipids can facilitate the secretion of reproductive hormones in aquatic animals. Jiang et al. observed that the dietary supplementation of 2.8% soybean lecithin resulted in a notable elevation in the E_2_ content in the ovary of *Monopterus albus* [57]. Ding et al. found that the addition of 4% soybean lecithin resulted in a notable increase in the ovarian index of *P. trituberculatus* and a concomitant elevation in the serum concentrations of VTG, P_4_, and E_2_ [51]. Yang also highlighted that phospholipid supplementation led to a notable elevation in the concentrations of 17β- E_2_ and P_4_ in ovarian tissue [58]. It is therefore hypothesized that 4.5% Antarctic krill oil can facilitate the conversion of steroids into sex steroid hormones in *M. rosenbergii*, thereby promoting vitellogenesis and ovarian development.

Ecdysone has been demonstrated to stimulate the proliferation of germline stem cells and to regulate vitellogenesis and ovarian development [59,60]. Ecdysone has been shown to be a hormone that initiates the synthesis of VTG in arthropods [61,62]. The genes *Nvd* and *dib* are involved in the synthesis of ecdysone [63,64]. *Nvd* is specifically expressed in tissues that synthesize ecdysone and is involved in cholesterol transport and the synthesis of steroid hormones [65]. Dib catalyzes 2,22-dideoxy-3-dehydroecdysone to form 2-deoxy-3-dehydroecdysone at C22 in the ecdysone synthesis pathway, thereby regulating the synthesis of ecdysone [66]. The highest expression levels are observed prior to and following the molting process, which coincides with elevated ecdysone levels [67]. As a nuclear receptor, *EcR* binds to ecdysone and activates downstream genes, such as *HR38*, *E75*, and *FTZ-F1*, to regulate vitellogenesis and oocyte maturation [68]. The present study demonstrated that Antarctic krill oil markedly elevated the secretion of ecdysone and significantly enhanced the expression of the ecdysone synthesis gene and signal transduction nuclear receptor gene. The K4 group demonstrated the most significant effect. It is proposed that Antarctic krill oil may influence ovarian development and vitellogenesis in *M. rosenbergii* by modulating the synthesis and signal transduction of ecdysone.

MF is a sesquiterpene hormone that plays a pivotal role in the reproductive process of crustaceans. It is synthesized by the mandibular organ and released into the hemolymph of crustaceans, and transported through the hemolymph to the hepatopancreas and ovary as a regulator of vitellogenesis [4]. Studies have shown that the hemolymph level of MF is maintained at a low level during the immature stage of crustaceans (I) and the initial phase of VTG synthesis (II), which results in a slower rate of ovarian development. During the rapid development of the ovary, the concentration of MF in the hemolymph increased to approximately three times of that observed in stages I and II [69]. One study demonstrated that the exogenous injection of MF can significantly enhance the OSI and oocyte volume of *Oziotelphusa senex senex*, thereby facilitating ovarian development [70]. In this study, it was found that the addition of 4.5% Antarctic krill oil led to a notable elevation in the concentration of MF in the hemolymph, accompanied by a substantial increase in the expression of Farnesoic acid-O-methyltransferase (*FAMeT*) mRNA and a reduction in the expression of *CYP15A1_C1*. In crustaceans, *FAMeT* is the rate-limiting enzyme for the synthesis of MF, which can catalyze the synthesis of MF from farnesic acid [71]. *CYP15A1_C1* is a specific isomer or variant of the *CYP15A1* gene, which functions as a catalyst for the degradation of MF [72]. The findings of this study suggest that Antarctic krill oil can facilitate the accumulation of MF in *M. rosenbergii* by accelerating the synthesis of MF and reducing the catabolism of MF. A noteworthy finding of this study is that the MF content in the K3 and K4 groups is approximately two- to three-times higher than that in the K1 group. This indicates that the administration of 3% to 4.5% of Antarctic krill oil can facilitate the transition of *M. rosenbergii* from the immature phase to the stage of rapid ovarian development, with the 4.5% krill oil supplementation demonstrating a superior effect.

Given that krill oil is rich in vitamin A, and retinol is a form of vitamin A, retinol is essential for female reproduction and may be involved in ovarian steroidogenesis, oocyte maturation, and corpus luteum formation [28]. A study demonstrated that the administration of 8000 IU/kg of retinol can induce a high expression of the *VTG* gene in the ovary and hepatopancreas by upregulating *VtgR*, the retinoid x receptor (RXR), ecdysone receptor (EcR), and ecdysone response gene E75. This results in an increase in the gonadal index of *Eriocheir sinensis* and the promotion of ovarian development [73]. It is worth noting that this study revealed that, in comparison to the control group, 4.5% Antarctic krill oil markedly elevated the expressions of *RDH12* mRNA, whereas no notable alterations were observed in the other groups. The levels of *ALDH* and *RXR* mRNA in each group that received Antarctic krill oil were significantly increased, with the level in the K4 group being the highest. *RDH12* is regarded as one of the retinol dehydrogenases (RDH) implicated in the vitamin A circulatory system [74], with the capacity to transform retinol into retinaldehyde. *ALDH* is an aldehyde dehydrogenase that utilizes retinal as a substrate, facilitating the conversion into retinoic acid [75]. In conjunction with the preceding report, the retinol metabolic pathway was found to be enhanced in the experiment involving the addition of krill oil [28,76,77]. It may therefore be posited that Antarctic krill oil exerts an influence on vitellogenesis and oocyte maturation through an increase in the expression of genes associated with retinol metabolism and an enhancement of the production and signal transduction of retinoic acid.

In general, reproductive hormones primarily regulate the transcriptional expression of *VTG* by binding to responsive receptors, with the *RXR-ECR* heterodimer potentially playing a pivotal role in this process. Retinoic acid, MF, and ecdysone have been demonstrated to play a role through *RXR* and *EcR* [76,77]. Similarly to ecdysone, it primarily binds to the EcR within the RXR-ECR heterodimer, subsequently recognizing and binding to specific hormone response elements (HREs) on the *VTG* gene, and ultimately binding to the heterodimer complex on the DNA as a transcription factor to initiate or enhance the transcription process of the *VTG* gene [78]. MF is considered to be a candidate ligand for RXR in crustaceans [79]. MF is capable of forming a heterodimer with *RXR*, namely *RXR-EcR*. The activation of the *RXR-EcR* heterodimer induces the expression of ecdysone genes (*E75* and *E74*) in the hepatopancreas, thereby promoting oocyte maturation and yolk production through ecdysone [76,80]. Retinoic acid or its derivatives binds to *RXR* as a ligand. *RXR* can directly bind to retinoic acid, but it is more common to form a heterodimer (*RXR-RAR*) with *RAR* [81,82]. Once *RXR-RAR* has bound to DNA, it is able to recruit co-activators or co-inhibitors, thereby regulating the transcription of downstream genes and regulating the growth, differentiation, and death of cells in the ovary.

## 5. Conclusions

In conclusion, the dietary supplementation of 4.5% Antarctic krill oil has been demonstrated to stimulate the synthesis and secretion of endocrine hormones (retinol metabolic factors, MF, sex steroid hormones, and ecdysone) in *M. rosenbergii*, thereby promoting vitellogenesis and oocyte maturation in this species. The findings of this study are of considerable importance for optimizing the feed formula of *M. rosenbergii* and enhancing ovarian development, thereby improving the reproductive rate, egg quality, and embryo development potential.

## Figures and Tables

**Table 1:** The formulation and proximate composition of the experimental diet used for *Macrobrachium rosenbergii*.

Ingredients (% Dry Matter)	K1	K2	K3	K4	K5
Fish meal	15	15	15	15	15
Cottonseed protein	10	10	10	10	10
Spray-dried blood cell powder	2	2	2	2	2
Soybean meal	20	20	20	20	20
Corn gluten meal	15	15	15	15	15
α-starch	22	22	22	22	22
Soybean oil	6	4.5	3	1.5	0
Antarctic Krill oil	0	1.5	3	4.5	6
Choline chloride	1	1	1	1	1
Vitamin–mineral premix ^1^	1	1	1	1	1
Bentonite	1	1	1	1	1
Calcium dihydrogen phosphate	2	2	2	2	2
Carboxymethyl cellulose	5	5	5	5	5
Total	100	100	100	100	100
Proximate analysis (%)					
Crude protein (%)	38.8	38.5	38.9	38.4	38.3
Ether extract (%)	8.22	8.19	8.20	8.23	8.24
Ash (%)	11.66	11.58	11.61	11.68	11.69

^1^ Premix supplied the following minerals (g·kg^−1^) and vitamins (IU or mg·kg^−1^):CuSO_4_·5H_2_O, 2.0 g; FeSO_4_·7H_2_O, 25 g; ZnSO_4_·7H_2_O, 22 g; MnSO_4_·4H_2_O, 7 g; Na_2_SeO_3_, 0.04 g; KI, 0.026 g; CoCl_2_·6H_2_O, 0.1 g; Vitamin A, 900,000 IU; Vitamin D, 200,000 IU; Vitamin E, 4500 mg; Vitamin K_3_, 220 mg; Vitamin B_1_, 320 mg; Vitamin B_2_, 1090 mg; Vitamin B_5_, 2000 mg; Vitamin B_6_, 500 mg; Vitamin B_12_, 1.6 mg; Vitamin C, 5000 mg; Pantothenate, 1000 mg; Folic acid, 165 mg.

**Table 2:** Primer sequences used for real-time PCR.

Gene	Forward (5′-3′)	Reverse (5′-3′)	Acquisition Pathway
*Vtg*	CCGACCATGCATTCACTCCGTTGA	TGTTGCCAAGGGACTTCAGTAGAGC	[29]
*VtgR*	TAGTCATAGTGGTGCTGCTCG	GAGAAGCGGTAAGTCTGGTT	[30]
*Cdc2*	TGCCTTGTAATCCTGTAGTTG	CCTCCCGATATTCTTGTCCT	TRINITY_DN104178
*Cyclin B*	ACATTCTGAGCGTCTGGTGC	ATGGCAAAGATGTCCTCTGTAGTT	TRINITY_DN1230
*RDH12*	ACGAACTCTATTCTGGCATCT	CAGCAAACAAATCGCCTACT	TRINITY_DN2519
*ALDH*	AGAGGCAATACGCAATACAC	CGAAGGTCAACAATGGGAAA	TRINITY_DN14240
*RXR*	GATCGGCAGTCCCCTTTGAA	TTGGACACACTGGGAGAAGC	[31]
*FAMeT*	GCACACTTGGCCCTCACTTC	CACACCACGTCGGGAGTTTC	[32]
*CYP15A1_C1*	TTCAGAGCGGCGACATTCAA	CAACGGTCAAAGGTGGGTCA	TRINITY_DN22898
*Met*	TGTGAAGAGGAGGCGGAGGA	AAGGCGAAGCGACTTGTGGT	TRINITY_DN12014
*nvd*	CATACCAGCCACATACACTT	TTGCTTGCCTTCATTACTCT	TRINITY_DN7487
*dib*	GGCATAGGAAAGAGTGAAGC	GTGGAGGCCAAAGATAGTGA	TRINITY_DN4119
*EcR*	AGAGCCGCATAAAGTGGAGA	CTCAGGTCGGTCAGGATGTT	[31]
*HR38*	TTAGGTGGAACAACAAGTGA	GATGGGTAATAACAGGCTTC	XM_067096834.1
*E75*	AGTTCCTCCGAGTCCTTATGTG	AGAATCGTCTGGGCTTTCAG	OQ626397.1
*FTZ-F1*	GGATCACCTGCACCAACGTA	GGAAACGATCTGCGAACTGC	[31]
*LDLR*	GGGCTATGCTCAACTGCTCG	AGGCAGGTTCCACTATGTGATGTA	TRINITY_DN5050
*17β-HSD1*	CGGCTGGAAATGCAGAAGTG	GATGTACTCGTCGCCGTAGG	[33]
*ERR*	AATACCAACGAACCACCCAA	GCTTCATCTCCGCACTCACT	TRINITY_DN100301
*18S*	GTCTGTGATGCCCTTAGATGTCC	GCAAGCCCCAATCCCTATC	[34]

**Table 3:** Growth performance of *Macrobrachium rosenbergii* fed different amounts of dietary Antarctic krill oil.

Parameters	IW/g	FW/g	WG/%	SGR/(%·Day^−1^)	SR/%
K1	4.59 ± 0.04	27.21 ± 2.34 ^a^	493.19 ± 51.79 ^a^	1.97 ± 0.09 ^a^	90.00 ± 2.88
K2	4.62 ± 0.10	29.69 ± 1.11 ^ab^	545.55 ± 37.59 ^a^	2.07 ± 0.06 ^a^	93.33 ± 3.33
K3	4.52 ± 0.05	36.22 ± 1.75 ^b^	702.62 ± 43.67 ^b^	2.31 ± 0.06 ^b^	93.33 ± 1.67
K4	4.51 ± 0.06	36.50 ± 3.00 ^b^	708.25 ± 58.69 ^b^	2.31 ± 0.08 ^b^	91.67 ± 3.33
K5	4.57 ± 0.09	32.40 ± 1.99 ^ab^	608.11 ± 32.94 ^ab^	2.17 ± 0.05 ^ab^	95.00 ± 4.58
ANOVA	0.806	0.030	0.017	0.013	0.782
Linear	0.517	0.022	0.016	0.010	0.337
Quadratic	0.577	0.028	0.018	0.015	0.476

Note: Values represent mean ± SEM (*n* = 4). Means in the same column with different superscripts are significantly different. Regression analysis using *p*-values.

**Table 4:** Effect of different levels of krill oil on the content of vitellogenin and reproductive hormones in the hemolymph of *Macrobrachium rosenbergii*.

Parameters	VTG (ng/mL)	EH (pg/mL)	E_2_ (pg/mL)	P4 (ng/mL)	MF (ng/mL)
K1	31.92 ± 1.28 ^a^	366.95 ± 18.7 ^a^	29.03 ± 1.09 ^a^	3.60 ± 0.26 ^a^	0.36 ± 0.05 ^a^
K2	41.44 ± 2.92 ^ab^	449.79 ± 24.86 ^bc^	38.25 ± 1.21 ^ab^	4.26 ± 0.43 ^ab^	0.51 ± 0.15 ^ab^
K3	47.30 ± 3.91 ^bc^	450.77 ± 29.91 ^bc^	49.05 ± 4.95 ^bc^	5.44 ± 0.43 ^b^	0.84 ± 0.15 ^c^
K4	62.97 ± 6.20 ^d^	498.62 ± 12.14 ^c^	70.20 ± 8.52 ^d^	5.64 ± 0.73 ^b^	0.93 ± 0.07 ^bc^
K5	55.94 ± 2.01 ^cd^	430.11 ± 12.24 ^b^	57.75 ± 6.93 ^cd^	4.93 ± 0.74 ^ab^	0.58 ± 0.10 ^abc^
ANOVA	<0.001	0.002	<0.001	0.025	0.015
Linear	<0.001	0.01	<0.001	0.007	0.061
Quadratic	0.093	0.002	0.119	0.053	0.007

Note: Each datum represents the mean of eight replicates. Data in columns assigned different superscripts are significantly different (*p* < 0.05). Regression analysis using *p*-values.

## Data Availability

Data are contained within the article.
